# miR-7a-5p Contributes to Suppressing NLRP3/Caspase-1 Signaling Pathway in Response to *Streptococcus suis* Type 2 Infection

**DOI:** 10.3390/microorganisms13081924

**Published:** 2025-08-18

**Authors:** Ziteng Deng, Qian Sun, Shun Li, Yibo Wang, Yuxin Che, Yunfei Huang, Jiedan Liao, Honglin Xie, Xiaoshu Zhan, Qinqin Sun, Qiang Fu

**Affiliations:** 1 School of Animal Science and Technology, Foshan University, No. 33 Guangyun Road, Foshan 528225, China; ztdeng@fosu.edu.cn (Z.D.); sunq202401@fosu.edu.cn (Q.S.); lishun@fosu.edu.cn (S.L.); wyb0652@fosu.edu.cn (Y.W.); yuxinche@fosu.edu.cn (Y.C.); yunfeihuang@fosu.edu.cn (Y.H.); jdliao@fosu.edu.cn (J.L.); honglinxie@nwafu.edu.cn (H.X.); xiaoshuzhan@fosu.edu.cn (X.Z.); sunqinqin@fosu.edu.cn (Q.S.); 2 Foshan University Veterinary Teaching Hospital, Foshan University, Foshan 528225, China; 3 Kunyu Integrated Agricultural Research Insititute, Xinjiang Academy of Agricultural and Reclamation Science, Shihezi 848116, China

**Keywords:** *Streptococcus suis* type 2, macrophage, NLRP3, miR-7a-5p, inflammation

## Abstract

*Streptococcus suis* type 2 (SS2) is a pathogen causing diseases like meningitis and septicaemia worldwide. While microRNAs (miRNAs) are acknowledged for their role in post-transcriptional regulation of gene expression and influence on immune responses, their exact functions in hosts during SS2 infection remain elusive. This study aims to explore the role of miR-7a-5p in macrophages during SS2 infection. Our findings reveal that SS2 infection in J774A.1 cells triggers upregulation of the NLRP3 inflammasome signaling pathways, evidenced by enhanced mRNA expression of pro-inflammatory cytokines (IL-6, IL-18, IL-23, TNF-α) and elevated protein levels of NLRP3, caspase-1, and IL-1β. Concurrently, SS2 infection reduces miR-7a-5p expression. Dual-luciferase reporter assays confirm that miR-7a-5p directly targets the 3′UTR of NLRP3 mRNA. Notably, miR-7a-5p overexpression in SS2-infected J774A.1 cells suppresses NLRP3 inflammasome activation and downstream signaling, as demonstrated by reduced mRNA levels of inflammatory mediators and decreased protein levels of NLRP3, caspase-1, IL-1β, and IL-18. Conversely, miR-7a-5p inhibition produces effects opposite to those of overexpression. In mice, administration of miR-7a-5p mimics mitigates SS2-induced lung, liver, and spleen damage, reducing histological scores in these organs. Collectively, these results show that miR-7a-5p alleviates SS2-induced inflammation by inhibiting the NLRP3 inflammasome, underscoring its potential as a therapeutic target for SS2-associated diseases.

## 1. Introduction

*Streptococcus suis* (*S. suis*), a globally distributed but often-underestimated Gram-positive pathogen, is zoonotic and can cause symptoms such as septicemia, arthritis, meningitis, and streptococcal toxic shock-like syndrome in infected hosts [1,2,3]. Based on capsular polysaccharide antigen polymorphism, *S. suis* has been classified into at least 56 serotypes, including authentic serotypes 1–19, 21, 23–25, 27–31, 1/2, Chz, and 26 novel capsular polysaccharide loci [1,4]. Among them, *S. suis* serotype 2 (SS2) is the most common and frequently reported isolate [1,5,6]. It not only causes significant economic losses in the swine industry but has also triggered three large-scale epidemics in humans in China [1,5]. However, the pathogenic mechanism of SS2 remains unclear, hindering effective prevention and eradication of SS2 infections.

MicroRNAs (miRNAs) are small non-coding RNAs that regulate gene expression by targeting specific sequences to modulate transcription, translation, or epigenetic modifications, which are essential for diverse physiological functions in organisms [7,8,9]. It is reported that miR-302b enhances host defense against bacteria by modulating inflammatory responses via TLR/IRAK4 feedback and suppresses pro-inflammatory cytokines by targeting phosphatase and tensin homolog [10]. In mice, miR-20b inhibits Mycobacterium tuberculosis-induced lung inflammation by targeting NLRP3 [11,12,13,14]. Our prior research found miR-223-3p inhibits NLRP3 inflammasome activation in *Streptococcus equi* ssp. *zooepidemicus* infection [15]. Over the past decade, miRNAs have proven essential to host–pathogen interaction, facilitating host defense yet enabling bacterial subversion of host functions [16,17]. Given the critical role of miRNAs in host antibacterial and anti-inflammatory responses, elucidating their specific mechanisms during SS2 infection is essential.

Macrophages, as pervasive cellular entities present in every tissue, function as the primary defense against pathogens [18,19]. When macrophages encounter bacteria, they activate numerous signal-transduction pathways that coordinate the antibacterial effectors to eliminate pathogens [20,21]. Additionally, as vital immunomodulatory factors, miRNAs regulate innate immune response by amplifying positive or negative feedback circuits triggered by pro-inflammatory and anti-inflammatory signals within macrophages [22,23]. Nevertheless, there is a dearth of research concentrating on the role of miRNA in macrophages during SS2 infection.

In this study, we aimed to explore the role of miR-7a-5p in macrophages during SS2 infection. When challenged with SS2, J774A.1 cells exhibited concurrent activation of the NLRP3 inflammasome pathway and suppression of miR-7a-5p expression. Notably, restoring miR-7a-5p levels via mimic transfection in both cellular and mouse models significantly inhibited SS2-triggered inflammatory responses. These findings establish miR-7a-5p as a critical negative regulator of NLRP3 inflammasome activity, thereby protecting macrophages from SS2-induced inflammation.

## 2. Materials and Methods

### 2.1. Bacterial Strains, Cell Lines and Mice

Bacterial and cell cultures, along with mouse husbandry, followed the established protocols detailed in prior publications [15].

The SS2 strain ZJ080101, initially isolated from diseased piglets and preserved at the China Institute of Veterinary Drug Control, was cultured in tryptone soy broth (OXOID, CM0131, Basingstoke, UK) or tryptone soya agar (OXOID, CM0129) at 37 °C under aerobic growth conditions. When required, the culture medium was enriched with 5% newborn calf serum.

J774A.1 murine macrophage-like cells (TIB-67, ATCC) were primarily used for SS2 infection experiments, while HEK-293T human embryonic kidney cells (CRL-3216, ATCC) were mainly employed for dual-luciferase reporter assays, with both cell lines maintained in Dulbecco’s Modified Eagle’s Medium (DMEM, Sigma, St. Louis, MO, USA) supplemented with 10% fetal bovine serum (FBS, HyClone, Logan, UT, USA), 1% Penicillin-Streptomycin Solution (HyClone), and 1% L-Glutamine (Sigma) under incubation at 37 °C in a 5% CO_2_-containing atmosphere [15].

Male BALB/c mice aged 6–8 weeks were obtained from the Laboratory Animal Center of Guangdong Province, China. All experimental procedures were rigorously reviewed and received approval for implementation from the Laboratory Animal Monitoring Committee of Guangdong Province.

### 2.2. J774A.1 Cells Infection Assay

The cell infection process was conducted following the methodology outlined in prior reports [24,25]. Upon reaching approximately 80% confluence, the cultured J774A.1 cells were transferred and seeded into 6-well plates. Following this, the cells were exposed to SS2 infection at a multiplicity of infection (MOI) of 10 for a 6 h period. Once the infection time elapsed, the cells were harvested. Western blotting analysis was performed to assess the expression levels of pertinent proteins, whereas quantitative reverse transcription polymerase chain reaction (qRT-PCR) was utilized to quantify the mRNA levels. Uninfected J774A.1 cells were included as the blank control group for comparison.

### 2.3. RNA Extraction and qRT-PCR Analysis

For the detection miR-7a-5p, total RNA was isolated from infected J774A.1 cells or mouse tissues utilizing the RNA simple Total RNA Kit (TIANGEN, Beijing, China). Subsequently, cDNA was synthesized using the miRcute Plus miRNA First-Strand cDNA Kit (TIANGEN). qRT-PCR was carried out on the LightCycler 480 (Roche, Basel, Switzerland) platform with the miRcute Plus miRNA qRT-PCR Kit (SYBR Green, TIANGEN), employing the reverse primer provided in the kit. The qRT-PCR protocol consisted of an initial incubation at 95 °C for 15 min, followed by 5 cycles of miRNA-specific enrichment (94 °C for 20 s, 65 °C for 30 s, 72 °C for 34 s), and then 40 cycles (94 °C for 20 s, 60 °C for 34 s) to proceed to the Melting Curve Stage. The relative expression of miR-7a-5p was determined using the 2^−ΔΔCT^ method, with U6 snRNA serving as the internal control and miR-7a-5p expression in control cells as the calibrator. To detect the expression levels of IL-6, IL-18, IL-23, and TNF-α, cDNAs were combined with primers and UltraSYBR Mixture (CWBIO, Boston, MA, USA) as per the manufacturer’s guidelines. The amplification process involved an initial step at 95 °C for 10 min, followed by 40 cycles of 94 °C for 30 s, 60 °C for 30 s, and 72 °C for 30 s. The CT values were quantified using the 2^−ΔΔCT^ method, with GAPDH as the reference gene for normalization. The primer sequences are provided in Table 1.

### 2.4. Western Blotting (WB)

Proteins were extracted from the relevant cells using a lysis buffer. Equal amounts of protein samples were prepared and subjected to separation through an SDS-PAGE assay (Solarbio, Beijing, China). Subsequently, the separated proteins were transferred onto PVDF membranes. The membranes were then blocked using a 5% bovine serum albumin solution. After washing, the membranes were incubated overnight at 4 °C with rabbit antibodies specific for mice, including anti-NLRP3 (ab263899, Abcam, Cambridge, UK), anti-caspase-1 (24232S, CST, Danvers, MA, USA), anti-IL-1β (ab283818, Abcam), anti-IL-18 (ab207323, Abcam), and anti-β-Actin (ab8227, Abcam). Following another round of washing, the membranes were incubated with secondary antibodies (Goat Anti-Rabbit IgG H&L, ab205718, Abcam) at room temperature for 1 h. Finally, the membranes were visualized and analyzed using a Gel Imaging System (Tanon, 4200, Shanghai, China).

### 2.5. Dual-Luciferase Reporter Assay

The dual-luciferase reporter assay was conducted in accordance with previously established protocols [15]. The potential miR-7a-5p binding site within the NLRP3 3′UTR was identified through bioinformatics tools, specifically TargetScan and miRDB. The specificity of this binding was validated by observing the targeting effect in the 3′UTR region that bound the miRNA, which was abolished upon mutation of that specific region. To ascertain whether NLRP3 mRNA is a direct target of miR-7a-5p, the predicted 431 bp miR-7a-5p binding sequence within the NLRP3 3′UTR (5′-CAUACCUUCAGCCUUGUCUUCCU-3′) or its mutant counterpart (5′-CAUACCUUCAGUCUUTGTGCTAU-3′) was cloned into the psiCHECK-2 vector (Sangon Biotech, Shanghai, China), resulting in the construction of the NLRP3–3′UTR-WT and NLRP3–3′UTR-Mut plasmids. HEK293T cells, grown to approximately 80% confluence, were seeded into 6-well plates and co-transfected with either 4 μg of the NLRP3–3′UTR-WT or NLRP3–3′UTR-Mut plasmid, along with 100 pmol of miR-7a-5p mimics (Sangon Biotech) or a negative control (NC, Sangon Biotech), using Lipofectamine 2000 (Invitrogen, Waltham, MA, USA). Thirty-six hours post-transfection, the cells were harvested, and luciferase activity was assessed using the Dual Luciferase Reporter Gene Assay Kit (Beyotime, Shanghai, China) on an Infinite 200 PRO NanoQuant (Tecan, Männedorf, Switzerland). Luciferase activity was normalized to Renilla luciferase activity to account for transfection efficiency. The relevant sequences are listed in Table 2.

### 2.6. Staining of Tissue Sections

The histological preparation of lung, liver, and spleen tissues adhered to our previously published protocol [25]. In brief, the tissues were fixed in 4% paraformaldehyde (PFA) for a duration of 48 h, subjected to dehydration through a series of graded ethanol and xylene treatments, and subsequently embedded in paraffin. The embedded tissues were sectioned into 8 μm-thick slices, stained with hematoxylin and eosin (H&E), and examined under a 40× objective lens using a Nikon Ni-U light microscope (Tokyo, Japan). Lung histopathology used three standardized parameters: edema severity (mild/moderate/extensive based on fluid patterns), inflammatory cell infiltration (graded minor/moderate/massive by leukocyte density per high-power field), and hemorrhage extent (classified by affected tissue area: <25%, 25–50%, or 50–75%). Each received a 4-point severity score (0 = absent, 1 = mild, 2 = moderate, 3 = severe). Liver assessment focused on lobular degeneration with focal necrosis (mild/moderate/marked by architectural distortion) and portal inflammation (graded similarly), scored 0–4 (0 = normal, 4 = severe remodeling with transitional scores 2–3). Splenic evaluation covered eight parameters, namely red pulp hyperplasia, sinus congestion, neutrophil infiltration, hemosiderin deposition, enlarged follicles, macrophage/plasma cell hyperplasia-each scored 0–3 based on tissue involvement (<20% = 1, 21–40% = 2, >41% = 3) [15].

### 2.7. Cell Transfection

For cell transfection, miR-7a-5p mimics, their corresponding negative control (NC), miR-7a-5p inhibitor, and its negative control (inhibitor NC) were procured from Sangon Biotech (Shanghai, China). To achieve overexpression of miR-7a-5p, J774A.1 cells, which had been cultured to 80% confluence in 6-well plates, were transfected with 50 nM of miR-7a-5p mimics or the miR-7a-5p mimics NC for a duration of 48 h using Lipofectamine 2000 (Invitrogen). For the knockdown of miR-7a-5p, cells at 80% confluence were transfected with 100 nM of miR-7a-5p inhibitor or the inhibitor NC for the same 48 h period, employing the same transfection reagent. Following transfection, the cells were infected with SS2 at a MOI of 10 for an additional 6 h and then collected for subsequent analysis.

### 2.8. Mouse Transfection

To investigate the potential involvement of miR-7a-5p in SS2-induced inflammation in a living organism, mice were administered in vivo-jetPEI-mediated miR-7a-5p through the tail vein, utilizing a previously established protocol [15,25]. Six-week-old BALB/c mice were randomly assigned to four distinct groups: Group I (control group, *n* = 6) received an intraperitoneal injection of PBS; Group II (*n* = 6) was injected with 10^4^ CFU of SS2; Group III (*n* = 6) received a combination of 10^4^ CFU of SS2 and 100 μL of in vivo-jetPEI-mediated miR-7a-5p mimics negative control (NC) (60 μg) via the tail vein; and Group IV (*n* = 6) was given 10^4^ CFU of SS2 along with 100 μL of in vivo-jetPEI-mediated miR-7a-5p mimics (60 μg) through the tail vein. Twenty-four hours post-treatment, the mice were euthanized, and their internal organs (lung, liver, and spleen) were collected. The organs were either fixed in 4% PFA or preserved at −80 °C for further analysis.

### 2.9. Statistical Analysis

For pairwise comparisons, two-tailed unpaired *t*-tests were utilized. In contrast, when analyzing data across multiple groups, a one-way analysis of variance (ANOVA) was conducted with the *p*-value threshold adjusted via the Bonferroni method to minimize the overall error rate, subsequently followed by Dunnett’s multiple comparisons test. Before performing these statistical analyses, the normality of the data distribution and the homogeneity of variances were evaluated. The results are reported as the mean ± standard error of the mean (SEM). Statistical significance was indicated by * *p* < 0.05, ** *p* < 0.01, *** *p* < 0.001, with ‘ns’ denoting non-significance. All statistical analyses were carried out using GraphPad Prism 9 software (GraphPad Software, San Diego, CA, USA).

## 3. Results

### 3.1. J774A.1 Cells Infected with SS2 Exhibited Up-Regulation of the NLRP3 Inflammasome and Downstream Pathways

**Figure 1:**
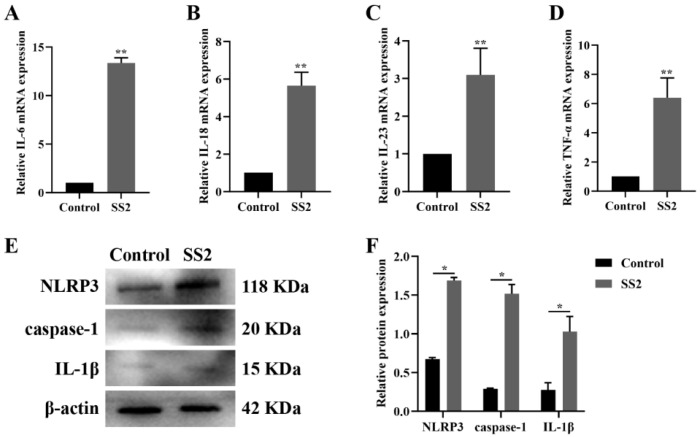
SS2 infection modulated the NLRP3/caspase-1 signaling pathway in J774A.1 cells. (**A**–**D**) qRT-PCR was used to detect the mRNA levels of IL-6, IL-18, IL-23, and TNF-α in both the Control group and the SS2 group. (**E**,**F**) WB analysis was performed to assess the expression levels of NLRP3, caspase-1, and IL-1β in both the Control group and the SS2 group. For qRT-PCR analysis, GAPDH was employed as the internal control for normalization. The mean fold changes in gene expression, relative to those in the Control group, were calculated using the 2^−ΔΔCT^ method. The data are presented as the mean ± SEM (*n* = 6 per group). Statistical significance is indicated as follows: * *p* < 0.05, ** *p* < 0.01.

The activation of the NLRP3 inflammasome triggers caspase-1-mediated proteolytic cleavage and subsequent activation of IL-1β and IL-18, thereby instigating an inflammatory cascade [26]. To determine whether SS2 could elicit an inflammatory response, we exposed J774A.1 cells to SS2 at a MOI of 10 for a duration of 6 h. The mRNA expression levels of inflammatory factors in J774A.1 cells were subsequently assessed using qRT-PCR analysis. Following SS2 infection, we observed a marked upregulation in the expression of IL-6, IL-18, IL-23, and TNF-α compared to the control group (Figure 1A–D). Additionally, SS2 infection resulted in a substantial increase in the protein levels of NLRP3 and caspase-1, which are key components of the NLRP3 inflammasome (Figure 1E,F). Furthermore, the protein level of IL-1β was significantly elevated after SS2 infection (Figure 1E,F). These findings indicate that SS2 infection can enhance the secretion of inflammatory cytokines and activate the NLRP3 inflammasome.

### 3.2. J774A.1 Cells Infected with SS2 Showed Down-Regulation of miR-7a-5p Expression

**Figure 2:**
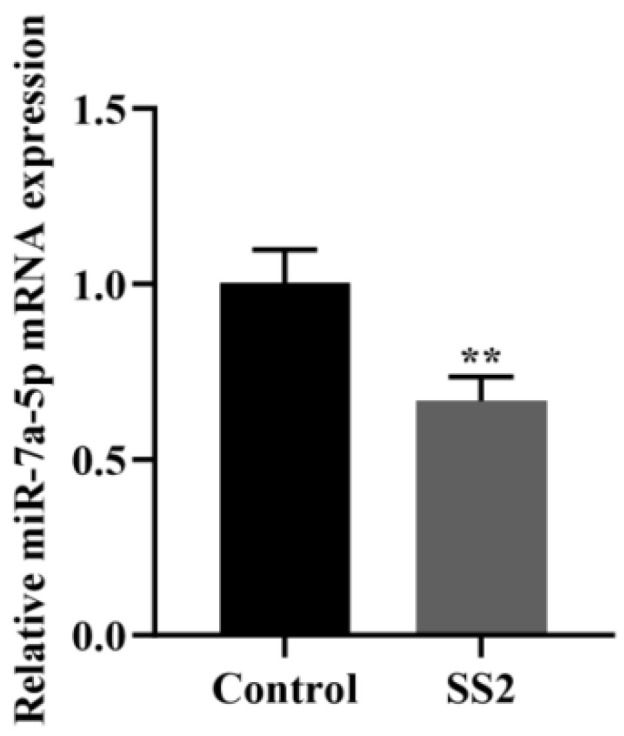
The expression of miR-7a-5p was inhibited in J774A.1 cells upon exposure to SS2. The expression level of miR-7a-5p was validated through qRT-PCR, where its levels were quantified relative to U6 snRNA and are presented as fold changes compared to the control samples. The data are presented as the mean ± SEM (*n* = 6 per group). Statistical significance is indicated as follows: ** *p* < 0.01.

qRT-PCR analysis was employed to evaluate changes in miR-7a-5p expression levels in J774A.1 cells after exposure to SS2. The results demonstrated that SS2 significantly downregulated miR-7a-5p expression (Figure 2).

### 3.3. miR-7a-5p Interacted with the 3′UTR Region of NLRP3 mRNA

**Figure 3:**
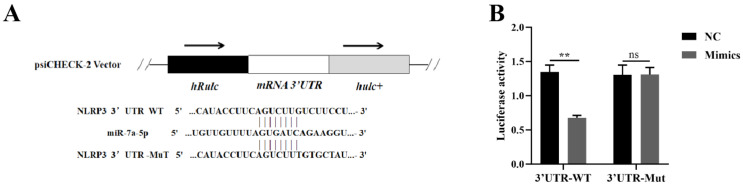
NLRP3 mRNA is a target of miR-7a-5p. (**A**) A diagram illustrates the predicted targeting sequences between miR-7a-5p and the 3′UTR of NLRP3 mRNA. (**B**) Luciferase activity was measured in 293T cells that were co-transfected with either the WT or Mut NLRP3-3′UTR constructs, along with miR-7a-5p mimics or their negative control (NC). The data are presented as the mean ± SEM (*n* = 6 per group). Statistical significance is denoted as follows: ** *p* < 0.01; ns: not significant. WT: wild-type; Mut: mutant-type; NC: miR-7a-5p negative control; mimics: miR-7a-5p mimics.

By leveraging the TargetScan and miRDB databases, we predicted that the 3′UTR of NLRP3 mRNA serves as a target for miR-7a-5p within host cells. To elucidate the functional mechanism of miR-7a-5p, we performed a plasmid-based dual luciferase reporter assay to ascertain whether NLRP3 mRNA is indeed a target of miR-7a-5p (Figure 3A). We cloned the sequences of the wild-type 3′UTR (3′UTR-WT) and the mutant 3′UTR (3′UTR-Mut) of NLRP3 mRNA into the psiCHECK-2 vector. Subsequently, we co-transfected these vectors into HEK293T cells along with miR-7a-5p mimics or a negative control (miR-7a-5p mimics NC). As illustrated in Figure 3B, miR-7a-5p significantly attenuated the luciferase activity associated with the 3′UTR-WT of NLRP3 mRNA. In contrast, it exerted no significant influence on the luciferase activity in cells transfected with the psiCHECK-2 vector containing the 3′UTR-Mut of NLRP3 mRNA, which harbored a mutation at the miR-7a-5p binding site. These findings conclusively demonstrate that NLRP3 mRNA is a direct target of miR-7a-5p.

### 3.4. miR-7a-5p Overexpression Suppressed the Activation of the NLRP3 Inflammasome and Its Downstream Pathways in Cells Infected with SS2

**Figure 4:**
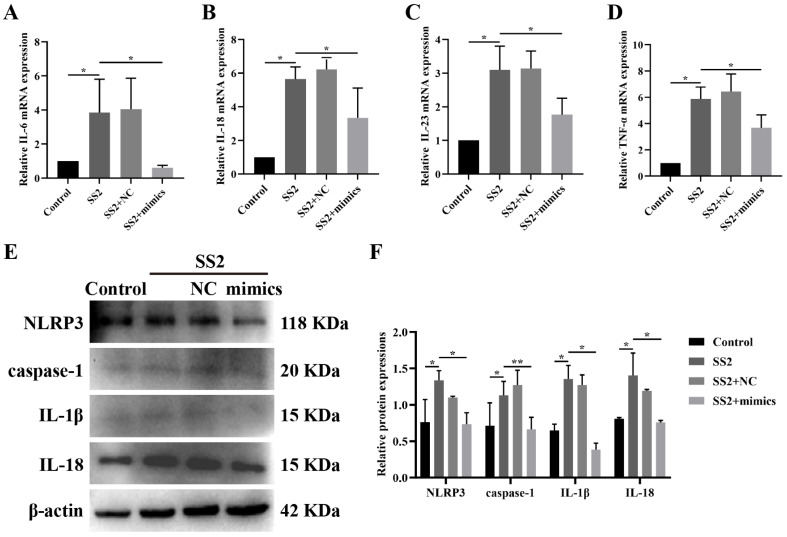
Overexpression of miR-7a-5p down-regulated the NLRP3/caspase-1 signaling pathway in J774A.1 cells during SS2 infection. (**A**–**D**) qRT-PCR was employed to detect the mRNA expression levels of IL-6, IL-18, IL-23, and TNF-α in the Control group, the SS2 group, the SS2+NC group, and the SS2+mimics group. (**E**,**F**) WB analysis was performed to assess the expression levels of NLRP3, caspase-1, IL-1β, and IL-18 in the Control group, the SS2 group, the SS2+NC group, and the SS2+mimics group. For qRT-PCR analysis, GAPDH was employed as the internal control for normalization. The mean fold changes in gene expression, relative to those in the Control group, were calculated using the 2^−ΔΔCT^ method. The data are presented as the mean ± SEM (*n* = 6 per group). Statistical significance is indicated as follows: * *p* < 0.05, ** *p* < 0.01.

To unravel the complex mechanism governing the impact of miR-7a-5p in SS2-stimulated J774A.1 cells, we transfected the cells with either miR-7a-5p mimics or a negative control (miR-7a-5p mimics NC), followed by SS2 stimulation for a duration of 6 h. Our findings revealed that the introduction of miR-7a-5p mimics markedly reduced the mRNA expression levels of IL-6, IL-18, IL-23, and TNF-α when compared to the group exposed solely to SS2 (Figure 4A–D). Furthermore, in J774A.1 cells transfected with miR-7a-5p mimics and subsequently infected with SS2, we observed a significant reduction in the levels of proteins associated with the NLRP3 inflammasome, particularly NLRP3 and caspase-1 (Figure 4E,F). Additionally, miR-7a-5p overexpression significantly curtailed the protein levels of IL-1β and IL-18 compared to the SS2-only group (Figure 4E,F). These results imply that miR-7a-5p overexpression effectively inhibits the activation of the NLRP3 inflammasome and its downstream signaling pathways in response to SS2 infection.

### 3.5. Blocking miR-7a-5p Boosted NLRP3 Inflammasome Activation and Downstream Pathways in SS2-Infected Conditions

**Figure 5:**
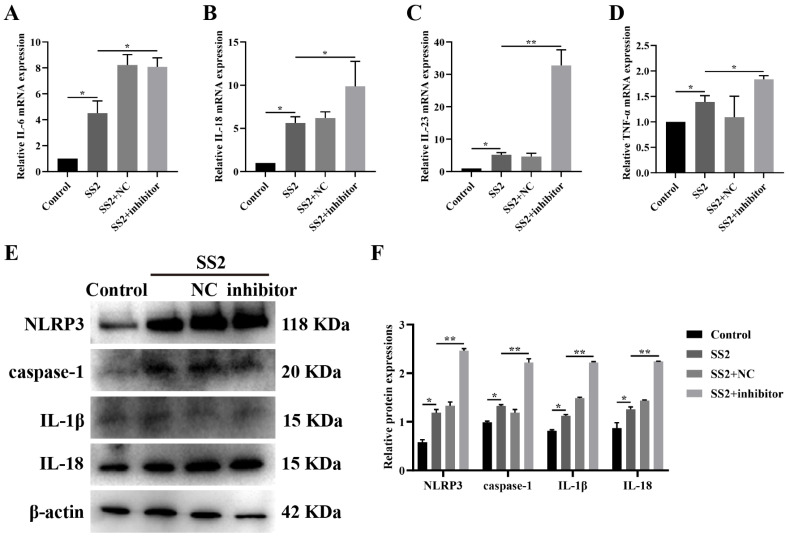
Inhibition of miR-7a-5p promoted the NLRP3/caspase-1 signaling pathway in J774A.1 cells during SS2 infection. (**A**–**D**) qRT-PCR was employed to detect the mRNA expression levels of IL-6, IL-18, IL-23, and TNF-α in the Control group, the SS2 group, the SS2+NC group, and the SS2+inhibitor group. (**E**,**F**) WB analysis was performed to assess the expression levels of NLRP3, caspase-1, IL-1β, and IL-18 in the Control group, the SS2 group, the SS2+NC group, and the SS2+inhibitor group. For qRT-PCR analysis, GAPDH was employed as the internal control for normalization. The mean fold changes in gene expression, relative to those in the Control group, were calculated using the 2^−ΔΔCT^ method. The data are presented as the mean ± SEM (*n* = 6 per group). Statistical significance is indicated as follows: * *p* < 0.05, ** *p* < 0.01.

To delve deeper into the mechanism through which miR-7a-5p influences SS2-stimulated J774A.1 cells, we pre-treated J774A.1 cells with a specific miR-7a-5p inhibitor prior to SS2 exposure. Our results indicated that, relative to the group exposed solely to SS2, the miR-7a-5p inhibitor caused a notable upregulation in the mRNA expression levels of IL-6, IL-18, IL-23, and TNF-α (Figure 5A–D). Furthermore, in the context of SS2 infection, the miR-7a-5p inhibitor significantly elevated the levels of proteins linked to the NLRP3 inflammasome, notably NLRP3 and caspase-1 (Figure 5E,F). Consistently, it also substantially augmented the protein expression of IL-1β and IL-18 when compared to the SS2-only group (Figure 5E,F). These observations imply that the inhibition of miR-7a-5p can amplify the activation of the NLRP3 inflammasome and its downstream signaling pathways in response to SS2 infection.

### 3.6. In Vivo Transfection with miR-7a-5p Mimics Alleviated Inflammation in Response to SS2

**Figure 6:**
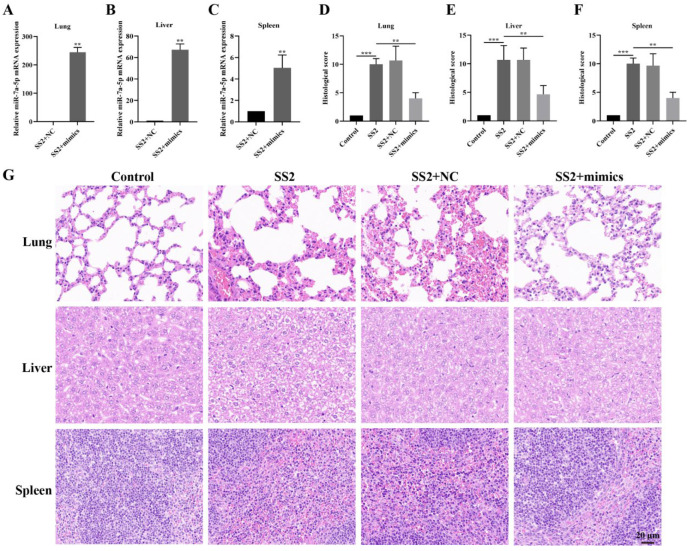
miR-7a-5p mitigated inflammation in mice triggered by SS2 infection. (**A**–**C**) The expression of miR-7a-5p was validated using qRT-PCR in the lung, liver, and spleen tissues. The relative expression levels of miR-7a-5p were determined by normalizing to the expression of U6 snRNA and are presented as fold changes compared to the NC samples. (**D**–**F**) Histological score was performed on tissue sections derived from the lung, liver, and spleen of mice. (**G**) Tissue sections from the lung, liver, and spleen of mice in the Control group, the SS2 group, the SS2+NC group, and the SS2+mimics group were stained using H&E. Scale bar, 20 μm. The data are presented as the mean ± SEM (*n* = 3 per group). Statistical significance is indicated as follows: ** *p* < 0.01, *** *p* < 0.001.

Based on the data we gathered, we initiated an in-depth investigation to assess the viability of employing miR-7a-5p mimics as a therapeutic approach for mice combating SS2 infection. To accomplish this, we administered miR-7a-5p, which was formulated with in vivo-jetPEI for efficient delivery, into mice through the tail vein, strictly adhering to a well-established protocol. Our findings revealed that the injection of miR-7a-5p mimics led to a marked and significant elevation in miR-7a-5p levels within the lungs, livers, and spleens of the mice (Figure 6A–C). In contrast, SS2 infection wreaked havoc on multiple organs, presenting as severe congestion and inflammatory exudate in the lungs, substantial hepatocyte swelling and hepatic sinusoid narrowing in the livers, as well as pronounced congestion and macrophage infiltration in the spleens (Figure 6G). Intriguingly, the administration of miR-7a-5p mimics to mice brought about a notable alleviation of the injuries observed in these organs (Figure 6G). Furthermore, the delivery of these mimics resulted in a substantial reduction in the elevated histological scores induced by SS2 infection (Figure 6D–F). Collectively, these results strongly imply that inhibiting the NLRP3 inflammasome through miR-7a-5p can effectively mitigate the inflammation triggered by SS2 infection.

## 4. Discussion

Despite extensive research efforts dedicated to unraveling the pathogenic mechanisms of SS2, the immune response mechanisms it elicits remain largely enigmatic. In this study, we primarily opted to infect J774A.1 cells with SS2 at a MOI of 10 for a duration of 6 h. This choice was informed by our preliminary experiments, which revealed that under these specific conditions, the cells remained viable without exhibiting any signs of floating or death, while concurrently displaying a significant upregulation in the expression of inflammatory cytokines. Building upon these findings, we further discovered that SS2 infection has the capacity to stimulate the secretion of key cytokines, including IL-1β, IL-6, IL-18, and TNF-α, and that this secretory response is intricately linked to the activation of the NLRP3 inflammasome within J774A.1 cells. NLRP3 inflammasomes are a cluster of protein complexes located within the cytoplasm, which assemble to coordinate the host immune responses against microbial infections and cellular damage [27,28]. This observation suggests that SS2 has the capacity to induce diseases linked to NLRP3 inflammasome activation. Previous studies have hinted that the NLRP3 inflammasome could serve as a promising therapeutic target for managing diseases associated with its dysfunction [29,30]. Moreover, prior research has underscored the pivotal role of the NLRP3 inflammasome in the processing and release of active forms of IL-1β and IL-18 [15]. In alignment with these existing findings, our results demonstrate that SS2 can significantly enhance the expression of IL-1β and IL-18. Consequently, more in-depth investigations are imperative to decipher the underlying regulatory mechanisms governing the release of IL-1β and IL-18 in response to SS2 infection.

In our recent investigation, we observed a substantial decline in miR-7a-5p expression following SS2 stimulation in J774A.1 cells, suggesting that miR-7a-5p may play a critical role in mediating the cellular response to SS2. While previous studies have underscored the essential role of miR-7a-5p in modulating NLRP3 inflammasomes [31], the exact molecular mechanisms underlying the interaction between SS2-induced NLRP3 inflammasome activation and miR-7a-5p remain poorly understood. To address this gap, we conducted a luciferase reporter assay, which confirmed that NLRP3 mRNA is directly targeted by miR-7a-5p in HEK-293T cells, with the binding site positioned within the 3′UTR. This finding implies that miR-7a-5p influences the regulation of NLRP3 translation, providing a potential mechanism for its involvement in the host response to SS2. NLRP3, a 115 kDa cytosolic protein, triggers the formation of a cytosolic multiprotein signaling complex—known as the NLRP3 inflammasome—upon activation [27,32]. The NLRP3 inflammasome is a critical multiprotein assembly that coordinates innate immune responses to infections by activating caspase-1 and promoting the maturation of key pro-inflammatory cytokines, namely pro-IL-1β and pro-IL-18 [15,26,30]. By synthesizing data from bioinformatic predictions and the results of the luciferase reporter assay, it is evident that miR-7a-5p functions as a central regulator of NLRP3 expression through post-transcriptional control, thereby offering new insights into the molecular interplay between host miRNAs and bacterial pathogen-induced immune responses.

NLRP3 functions as a critical mediator of inflammatory responses within macrophages, playing an indispensable role in innate immune reactions [30]. Upon bacterial invasion, the NLRP3 inflammasome is activated, leading to the robust production of pro-inflammatory cytokines, including IL-6, IL-18, TNF-α, and IL-23 [26]. While an appropriate inflammatory response is essential for host defense against bacterial pathogens at the site of infection, an excessive inflammatory response can result in exacerbated tissue damage. Consequently, precise regulation of inflammatory cytokine production is vital during bacterial infections. Additionally, miRNAs are integral to an effective immunological response, contributing to the control and clearance of infections [33,34]. To thoroughly assess the impact of miR-7a-5p on the inflammation induced by SS2 in J774A.1 cells, we transfected miR-7a-5p mimics and their negative controls into these cells. Experimental results revealed that miR-7a-5p mimics significantly attenuated SS2-induced overexpression of pro-inflammatory cytokines while concurrently suppressing NLRP3 signaling pathway, demonstrating that miR-7a-5p exerts anti-inflammatory effects in SS2-induced inflammation. To comprehensively validate this mechanism, we further transfected J774A.1 cells with an miR-7a-5p inhibitor and observed effects opposite to those of miR-7a-5p mimics. This reverse experimental outcome provides robust evidence that miR-7a-5p can exert anti-inflammatory effects in SS2-induced inflammation in J774A.1 cells.

Currently, the therapeutic potential of miRNA delivery for treating various diseases has been extensively documented [35,36,37], and our study provides further compelling evidence supporting this potential. Upon administering miR-7a-5p mimics via tail vein injection using in vivo-jetPEI as the carrier, we observed a significant and notable upregulation in miR-7a-5p expression levels within the lungs, liver, and spleen. Furthermore, to assess the safety profile of the transfection process, our preliminary trial results revealed that when mice were administered the transfection reagent jetPEI alone, they exhibited no abnormal symptoms in comparison to normal mice. This observation unequivocally indicates not only the effective delivery of these miR-7a-5p mimics to multiple tissues and organs but also highlights the biocompatibility and safety of the in vivo-jetPEI delivery system. Subsequent in-depth morphological examinations further elucidated that, in the lungs, miR-7a-5p mimics exerted a pronounced effect by significantly curtailing inflammatory cell infiltration, alleviating alveolar damage, and preserving a relatively intact lung tissue structure that closely resembled the morphological characteristics of the control group. This observation fully substantiates their mitigating impact on lung injury in mice induced by SS2 infection. Moving to the liver, the mimics markedly attenuated liver damage resulting from SS2 infection, as evidenced by a reduction in inflammatory cell infiltration within liver tissue and a diminished area of hepatocyte necrosis, strongly implying their efficacy in alleviating liver injury in SS2-infected mice. Furthermore, in the spleen, treatment with miR-7a-5p mimics facilitated the restoration of spleen morphology and structure, while concurrently leading to a significant reduction in spleen enlargement, inflammatory response, and tissue damage scores. Although our research primarily focuses on murine models and cellular experiments, studies confirm that occupational exposure to SS2-infected pigs significantly increases the risk of shock and inflammatory complications [38]. Importantly, we demonstrate that miR-7a-5p reduces SS2-induced inflammation through specific modulation of NLRP3 pathways. This work provides a scientific basis for developing novel therapeutic strategies targeting porcine and human SS2 infections, while offering translational potential to mitigate infection risks.

However, we must acknowledge the limitation of the current study. Our experimental design relied solely on the J774A.1 macrophage line, which may restrict the translational applicability of our findings. To address this, subsequent investigations should employ a broader array of cellular models, including MH-S alveolar macrophages and primary macrophages isolated from bronchoalveolar lavage fluid, thereby enhancing the robustness and generalizability of the conclusions. In our in vivo mouse studies, administration of miR-7a-5p mimics may concurrently modulate targets beyond NLRP3, including NLRP1, NLRC4, GSDMD, and caspase-1. Critically, the regulatory effects may not be confined solely to macrophage NLRP3 but rather extend to NLRP3 in other cell types, including neutrophils and epithelial cells. Thus, parallel in vivo studies incorporating miR-7a-5p inhibitors are warranted for comprehensive validation of both on-target and off-target regulatory effects. Given the structural similarity between microRNAs and mRNAs, miR-7a-5p mimics delivered via in vivo jetPEI are susceptible to degradation by serum RNases. Additionally, jetPEI-mediated delivery often results in systemic distribution of miR-7a-5p mimics beyond target organs (lungs, spleen, and liver), potentially inducing unintended systemic immune responses. In vivo mouse experiments necessitate simultaneous evaluation of inflammatory marker expression in lungs, liver, and spleen alongside quantification of SS2 bacterial load, thereby providing robust evidence for the anti-inflammatory efficacy of miR-7a-5p.

## 5. Conclusions

**Figure 7:**
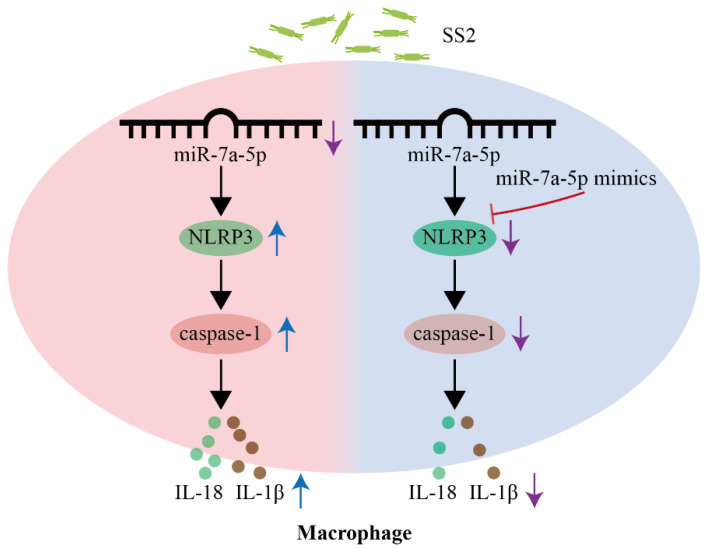
Proposed model by which miR-7a-5p attenuates SS2-induced inflammation through inhibiting NLRP3 inflammasome/caspase-1 signaling pathway.

SS2 is recognized as a zoonotic threat endangering human and animal health, whereas miRNAs profoundly modulate host–pathogen interactions. Our current research findings reveal that miR-7a-5p is involved in inhibiting the activation of the NLRP3 inflammasome during SS2 infection. Notably, miR-7a-5p has the ability to mitigate inflammation triggered by SS2, both in mice and in J774A.1 cells. These discoveries offer fresh perspectives and point to miR-7a-5p as a promising therapeutic target for addressing conditions associated with SS2 (Figure 7).

## Figures and Tables

**Table 1:** Primers for cytokines.

Name	Sequence (5′-3′)
IL-6	F: TAGTCCTTCCTACCCCAATTTCC R: TTGGTCCTTAGCCACTCCTTC
IL-18	F: GTGAACCCCAGACCAGACTG R: CCTGGAACACGTTTCTGAAAGA
IL-23	F: CCCGTATCCAGTGTGAAGATG R: GGCTCCCCTTTGAAGATGTC
TNF-α	F: CCTGTAGCCCACGTCGTAG R: GGGAGTAGACAAGGTACAACCC
GAPDH	F: TGACAACAGCCTCAAGATCG R: GTCTTCTGGGTGGCAGTGAT
U6 snRNA	F: CTCGCTTCGGCAGCACA
miR-7a-5p	F: GCGCGTGGAAGACTAGTGATTT

**Table 2:** Sequences for miR-7a-5p.

Name	Sequence (5′-3′)
miR-7a-5p mimics	F: UGGAAGACUAGUGAUUUUGUUGU R: ACAACAAAAUCACUAGUCUUCCA
miR-7a-5p mimics negative control, NC	F: UCGCGCGACACCGCUAGCUAG R: AGCUAGCGGUGUCGCGCGAUU
miR-7a-5p inhibitor	ACAACAAAAUCACUAGUCUUCCA
miR-7a-5p inhibitor negative control, inhibitor NC	CUAGCUAGCGGUGUCGCGCGA

## Data Availability

The original contributions presented in this study are included in the article. Further inquiries can be directed to the corresponding author.

## Abbreviations

The following abbreviations are used in this manuscript:

SS2	*Streptococcus suis* type 2
NLRP3	NOD-like receptor family pyrin domain containing 3
IL-1β	Interleukin-1 beta
IL-6	Interleukin-6
IL-18	Interleukin-18
TNF-α	Tumor necrosis factor α
